# 499. Seeing the full picture: Adding discharge antibiotic durations to the dashboard

**DOI:** 10.1093/ofid/ofae631.151

**Published:** 2025-01-29

**Authors:** Elizabeth Dodds Ashley, April Dyer, Jeannette Bouchard, Melissa D Johnson, Angelina Davis, Deverick J Anderson, Deverick J Anderson, Rebekah W Moehring

**Affiliations:** Duke Center for Antimicrobial Stewardship and Infection Prevention, Durham, NC; Duke Center for Antimicrobial Stewardship and Infection Prevention, Durham, NC; Duke Antimicrobial Stewardship Outreach Network, Elgin, SC; Duke University, Durham, North Carolina; Duke Center for Antimicrobial Stewardship and Infection Prevention, Durham, NC; Duke Center for Antimicrobial Stewardship and Infection Prevention, Durham, NC; Duke Center for Antimicrobial Stewardship and Infection Prevention, Durham, NC; Duke University, Durham, North Carolina

## Abstract

**Background:**

Antibiotic use (AU) at hospital discharge is often inappropriate and can account for the majority of antibiotic exposure attributed to an admission. Tracking and optimizing discharge antibiotic prescriptions (Rx) can be challenging for antimicrobial stewardship programs (ASPs), as inpatient and outpatient AU are not linked in many electronic health records. Our aim was to establish a routine metric for total antimicrobial durations across a network of hospitals comprising Duke Antimicrobial Stewardship Outreach Network (DASON) and Duke University Health System (DUHS).

**Figure:**
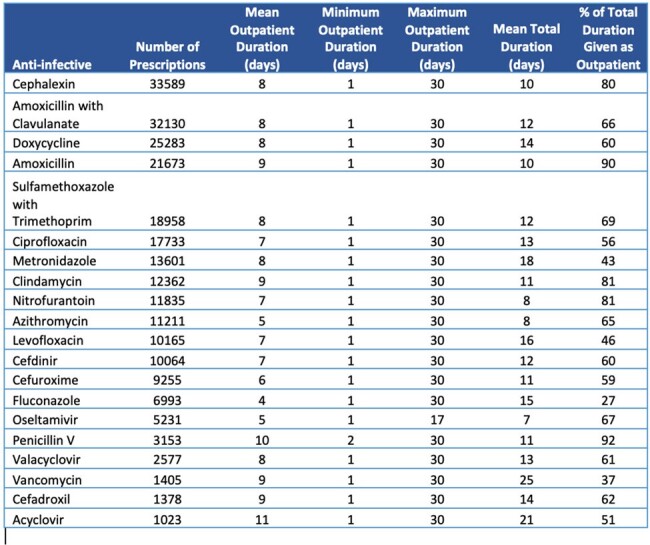

**Methods:**

We created a data extract for discharge antibiotic Rx and incorporated this into existing data infrastructure supporting DASON and DUHS hospital-based ASPs. Extracts included discharge antibiotic Rx for agents within the American Hospital Formulary Service (AHFS) category for anti-infectives (8.00). Administration instructions and quantity were used to calculate antibiotic days of therapy (DOT) by agent for discharge Rx. Exclusion criteria were: 1) antiretroviral and anti-COVID medications, 2) Rx that had a paired discontinuation order within the first 24-48 hours 3) Rx with a duration > 30 days, 4) Rx where durations could not be calculated, and 5) non-enteral medications. The clinical reason for use was inferred from most proximal inpatient AU order if prescriber entered indication was available. Total antibiotic duration was determined by adding inpatient DOT and calculated discharge Rx DOT.

**Figure:**
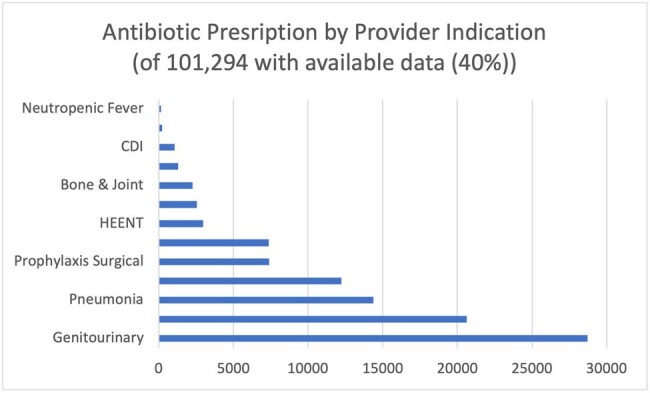

**Results:**

Data from calendar year 2023 was available for 20 hospitals in DASON/DUHS. Of the initial 307,825 Rx identified, 255,543 Rx in 194,428 patients remained after applying exclusion criteria. Of interest, 83,276 (27%) patients received a discharge antibiotic Rx despite no record of inpatient AU. Data for the top 20 agents with a discharge Rx are shown in the Table; narrow spectrum agent use was common. Much of the total antibiotic course was prescribed post-discharge (range 27-90%). Genitourinary and skin/soft tissue infection (SSTI) were the most common Rx indications (Figure).

**Conclusion:**

Antibiotics prescribed at discharge remains an important stewardship target. Data regarding AU at discharge can be combined with inpatient AU for routine monitoring to facilitate ASP interventions at TOC.

**Disclosures:**

**Elizabeth Dodds Ashley, PharmD, MHS**, HealthtrackRx: Advisor/Consultant|UpToDate: Royalties **Melissa D. Johnson, PharmD MHS AAHIVP**, Biomeme: Licensed Technology|Scynexis, Inc: Grant/Research Support|UpToDate: Author Royalties **Rebekah W. Moehring, MD, MPH, FIDSA, FSHEA**, UpToDate, Inc.: Author Royalties

